# Protective Effect of Royal Jelly on *In Vitro* Fertilization (IVF) in Male Mice Treated with Oxymetholone

**DOI:** 10.22074/cellj.2015.19

**Published:** 2015-10-07

**Authors:** Ensieh Zahmatkesh, Gholamreza Najafi, Vahid Nejati

**Affiliations:** 1 Department of Histology and Embryology, Faculty of Science, Urmia University, Urmia, Iran; 2 Department of Anatomy and Embryology, Faculty of Veterinary Medicine, Urmia University, Urmia, Iran

**Keywords:** Catalase, Embryo, Fertilization, Oxymetholone, Royal Jelly

## Abstract

**Objective:**

This study aimed to investigate the effects of royal jelly (RJ) on catalase, total antioxidant capacity and embryo development in adult mice treated with oxymetholone (OXM).

**Materials and Methods:**

In this exprimental study, 32 male and 96 female adult Naval Medical Research Institute (NMRI) mice (7-9 weeks of age) with a ratio of 1:3 for fertili- zation purposes were randomly divided into 4 groups as follows: i. Control group (n=8) receiving 0.1 ml/mice saline daily by gavage for 30 day, ii. RJ group (n=8) treated with RJ at a dose of 100 mg/kg daily by gavage for 30 days, iii. OXM group (n=8) receiving OXM at the dose of 5 mg/kg daily by gavage for 30 days and iv. RJ+OXM group (n=8) receiving RJ at the dose of 100 mg/kg daily by gavage concomitant with 100 mg/kg OXM adminis- tration for 30 days.

**Results:**

Analysis revealed a significant reduction in catalase, total antioxidant, as well as embryo development in OXM group (P<0.05). However, RJ group showed a salient recovery in the all of the above mentioned parameters and embryo toxicity.

**Conclusion:**

The results of this study indicated a partially protective effect of RJ against OXM-induced embryo toxicity.

## Introduction

Infertility is a fairly common problem and defined by World Health Organization (WHO) failure to conceive after 12 months of unprotected intercourse (1, 2). Infertility has different aspects including psychological and interpersonal stress which severely affects infertile couples. Globally, ranging between 8 and 12% among women of childbearing age, infertility affects 50-80 million people. Some of its aspects important roles in Eastern countries and, become a psychosocial burden (3, 4). A substantial share of the total infertility falls on males (5, 6). Oxymetholone (OXM) is considered as a synthetic derivative of testosterone, made by methylation of 17-alpha carbon and saturation of 5-alpha carbon, and synthesized for the first time by Ringold in 1959 (7). Anabolicandrogenic steroids (AAS), particularly OXM, are used at small doses (1-5 mg/Kg) for treating anemia, failure to thrive (FTT) in children, and heart failure (8). This type of drug increases erythropoietin production that is followed by affecting bone marrow directly to increase hemoglobin level and red blood cell count (9). Additionally, OXM can promote synthesis of protein, nitrogen retention and deposition of calcium in bones (10). In order to assume anabolic properties, these steroids must be used at 10-100 times their normal (therapeutic) doses in which their adverse reactions occur, (11) having profound effects on male endocrine and reproductive systems. It has been found that AASs-induced low male infertility can be reversible. Also, researches have indicated that persistent hypogonadotrophic hypogonadism caused by steroid abuse could be resolved after the AAS withdrawal (12). Royal jelly (RJ) is a milky white highly viscous substance secreted from the salivary gland of the honeybee worker, Apismellifera (Apidae), which is essential in the development of queen bees (13). This substance possesses many physical and chemical properties, including anti-inflammatory, antioxidant, anti-tumor, and immunomodulatory functions in experimental animals (14), which are beneficial to human health, leading to its wide use in commercial and medical products, health food, and cosmetics (15). *In vitro* fertilization (IVF), first successfully applied in 1978, is the last option for infertile couples who failed to conceive. In this international method, different standard therapies like surgery, fertility drugs and artificial insemination are applied to treat infertility cases like male and immunological infertility, infertility caused by endometriosis, and other unexplained conditions (16, 17). This study aims to investigate the effects of RJ on catalase, total antioxidant capacity and embryo development in adult mice treated with OXM.

## Materials and Methods

### Animal model

In this exprimental study, two groups of 7-9- week old adult Naval Medical Research Institute (NMRI) mice, 32 male and 96 female, weighing 30 ± 2 g and 27 ± 2 g, respectively, were purchased from the Animal House of Science Faculty, Urmia University, Urmia, Iran. All animals were allowed free access to water and food ad libitum in controlled conditions of temperature (22 ± 2˚C), relative humidity (55 ± 5%) and normal photoperiod.

### Drugs

OXM was used at a dose of 5 mg/kg (pilot). Drug was dissolved in saline before oral administration. RJ was used at the dose of 100 mg/kg (18).

### Experimental protocol

In this investigate, 32 male and 96 female with a ratio of 1:3 for fertilization purposes were divided into following 4 groups: i. control group (n=8) receiving 0.1 ml/mice saline daily by gavage for 30 days, ii. OXM group (n=8) treated with 5 mg/kg OXM daily by gavage for 30 days, iii. RJ group (n=8) treated with 100 mg/kg RJ daily by gavage for 30 days and iv. RJ+OXM (n=8) group treated with 5 mg/kg OXM by gavage along with 100 mg/ kg RJ daily for 30 days. All experimental procedures were approved by the Ethics Committee for Animal Experimentation of Urmia University, Urima, Iran.

### Mouse preparation for in vitro fertilization (Harvesting of oocytrs)

Each female mouse was injected subcutaneously with 10 IU pregnant mare’s serum gonadotropin (PMSG, Boxmeer, Netherlands) 48 hours prior to receiving an intraperitoneal injection of 10 IU human chorionic gonadotropin (hCG, Folligon, Netherlands). The animals were euthanized by dislocation of cervical vertebrae, 12-14 hours after hCG administration. Their ampulla of oviducts were removed and transferred to a petri dish that contained 1 ml human tubular fluid (HTF, Sigma, St. Louis, USA) medium+4 mg/ml bovine serum albumin (BSA, Sigma, St. Louis, USA). Using a stereo microscope (Model TL2, Olympus Co., Tokyo, Japan), the ampullary portion of fallopian tube was found and oocytes were dissected out.

### Preparation of culture media for in vitro fertilization

One day before conception, required media were prepared for fertilization and incubated 5% CO_2_ and 37˚C for 12 hours. For each group, separate conception dishes were considered containing 1 ml HTF medium combined with 4 mg/ml BSA. A drop of 500 ml and a few drops of 100 ml per dish were used for washing the IVF dishes and they were then covered with mineral oil (Sigma, St. Louis, USA).

### Sperm preparation

Male mice were euthanized by dislocation of cervical vertebrae to prepare needed sperm. Then, the skin of the abdomen was sterilized with 70% ethanol (Merck, USA) and after making an incision in the abdomen and removing the surrounding connective tissue, caudal epididymis was isolated from testes and placed in a petri dish containing 1 ml HTF medium combined with 4 mg/ml BSA which had reached equilibrium before. After making several cuts in the tail of the epididymis and using pressure in vas deferens, sperm output was placed in an incubator at 5% CO_2_ and 37˚C for 30 minutes. Spermatozoa were then spread out in the medium.

### Ovulation and fertilization in the in vitro fertilization laboratory

Between 12 and 14 hours after injection of hCG (in the morning after), female mice were euthanized by dislocation of cervical vertebrae, and the ampule of fallopian tubes were then removed and put in the HTF medium at 37˚C. Using dissection techniques, oocytes were removed, washed with the HTF, and transferred to the fertilization droplets under mineral oil containing HTF+BSA. Following capacitation step, sperms (1×10^6^/1 ml HTF) were added to the medium, one million per ml of culture. Fertilization is determined about 4 to 6 hours after releasing the sperm by observing two pre-nucleus. After granulosa cells were denuded and washed, these zygotes were transferred into the fresh pre-equilibrated medium and cultured for five days.

### Assessment of two-cell embryos growth

Evaluation of two-cell embryos was done 24 hours after fertilization. The percentage of blastocyst- stage embryos was performed on days 4 and 5 of fertilization.

### Measurement of catalase activity

Catalase activity was determined based on its ability to decompose hydrogen peroxide (H_2_O_2_) in homogenized testicular tissue using the method of Aebi (19). Decomposition of H_2_O_2_ is considerable by reducing the absorption at 240 nm in an absorption spectrum. For this purpose, 30 mM H_2_O_2_ (Merck, USA) as substrate and 50 mM phosphate buffer solution (PBS, PH=7, Merck, USA) were used as an alternative substrate in the blank solution. The testis tissue pieces were homogenized in PBS and then centrifuged (Eppendorf AG 5810R, Germany) at 3400 rpm for 15 minutes. Subsequently 1 ml H_2_O_2_ was added to 2 ml supernatant and absorbance was measured at 240 nm in a spectrophotometer (pharmaciaп novaspec, and biochrom, England). The values were expressed in terms of U/g tissue.

### Measurement of total antioxidant ferric reducing antioxidant power test

For automatic measurement and evaluation of antioxidants, the ferric reducing antioxidant power (FRAP) was applied. Reduction of ferric to ferrous ions in low PH forms a colored complex of ferrous-tripyridyltriazine (FeIITPTZ). Reactive oxygen species (ROS) that are potentially harmful are produced as byproducts of normal aerobic metabolism and usually removed or disabled by antioxidants groups in in vivo. In low PH, ferric tripyridyltriazine (FeШ- TPTZ) complex is reduced to form the Feп and to produces an intense blue color with a high absorption at 593 nm. FRAP reagent (with a ratio of 10:1:1) including 25 ml of (300 mM PBS, 10 mM TPTZ in 40 mM HCL, Merck, USA) and 2.5 ml of 0.0540 g FeCl3 (Merck, USA) were poured into a flask and brought to volume of 10 ml with distilled water sample was weighted dissolved into 1.5 M of 10% (W/V) KCL buffer (Merck, USA) and grounded in a mortar. The solution was then poured in a micro-tube and centrifuged at 1000 rpm for 5 minutes. One hundred ml of supernatant was poured into a test tube, mixed with 3 mL of FRAP reagent and incubated in water bathat 37˚C for 7-10 minutes. The absorbance of blue complex was then determined at 593 nm. Data are expressed in mmol tissue weight (FRAP value) (20, 21).

### Statistical analysis

All data were analyzed using the one-way ANOVA by Tukey-Kramer test. The level of significance was considered at P<0.05. All analysis for each sample were done individually.

## Results

### Catalase concentration

Results show that OXM caused a significant decrease in catalase enzyme compared to the control group (P<0.05). However RJ+OXM group partially compensated this lack attributed to the OXM group (Table 1).

### Total antioxidant concentration (FRAP test)

In the OXM group, total antioxidant concentration dropped as compared with the control group, while in the RJ group, there was a significant increase in total antioxidant concentration in comparison with the control group (P<0.05). The OXM+RJ showed a significant increase in this regard as compared with the control and OXM group (P<0.05, Table 1).

### Fertilization and embryonic development

In this study, OXM caused a significant decrease in fertilization rate and percentage of blastocysts (P<0.05) and a significant increase in the percentage of arrested embryos (P<0.05), but did not affect percentage of the two-cell embryos. RJ caused an increase in fertilization rate and percentage of blastocyst and a decrease in percentage of the arrested embryos (P<0.05) and two-cell embryo (Table 2, Figes.1, 2).

**Table 1:** Effects of OXM, RJ as well as OXM and RJ on catalase activity and FRAP level in adult male mice


Groups	Catalase (U/g tissue)	FRAP (mMol/g tissue)

Control	0.516 ± 0.060	126.66 ± 0.927
OXM	0.230 ± 0.026^a^	103.33 ± 4.371
RJ	0.530 ± 0.060^b^	135.43 ± 7.361^b^
OXM+RJ	0.320 ± 0.020	214.33 ± 8.685


Values are expressed as mean ± standard deviation (n=8). ^a^; Significant differences as compared with the control group (P<0.05), ^b^; Significant differences as compared with the OXM group (P<0.05), OXM; Oxymetholone, RJ; Royal jelly and FRAP; Ferric reducing antioxidant power.

**Table 2:** Effect of OXM , RJ as well as OXM and RJ on fertilization and embryonic development rates


Groups	Oocyte (n)	Zygote (n/%)	Two cell (%)	Blastocyst (%)	Arrest (%)

Control	38	(33) 86.72 ± 1.92	78.48 ± 2.89	73.04 ± 0.71	14.65 ± 1.00
OXM	47	(32) 67.81 ± 1.55^a^	77.18 ± 4.34	40.64 ± 2.02^a^	27.17 ± 3.58^a^
RJ	85	(76) 89.32 ± 0.41^b^	88.71 ± 2.64	82.40 ± 2.10^b^	6.92 ± 2.51^b^
OXM+RJ	58	(44) 75.76 ± 2.47	63.43 ± 1.92	60.51 ± 1.20^b^	15.24 ± 1.27^b^


Values are expressed as mean ± standard deviation (n=8). ^a^; Significant differences as compared with the control group (P<0.05), ^b^; Significant differences as compared with the OXM group (P<0.05), OXM; Oxymetholone and RJ; Royal jelly.

**Fig.1:**
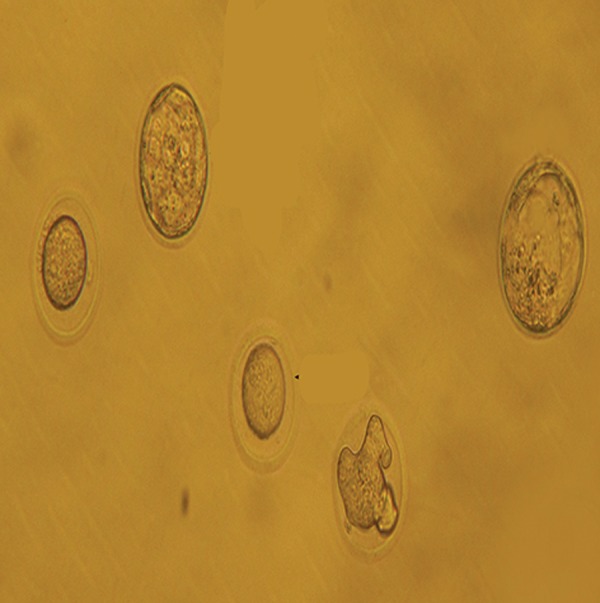
Fertilized and infertilized oocytes in OXM group (×200). OXM; Oxymetholone.

**Fig.2:**
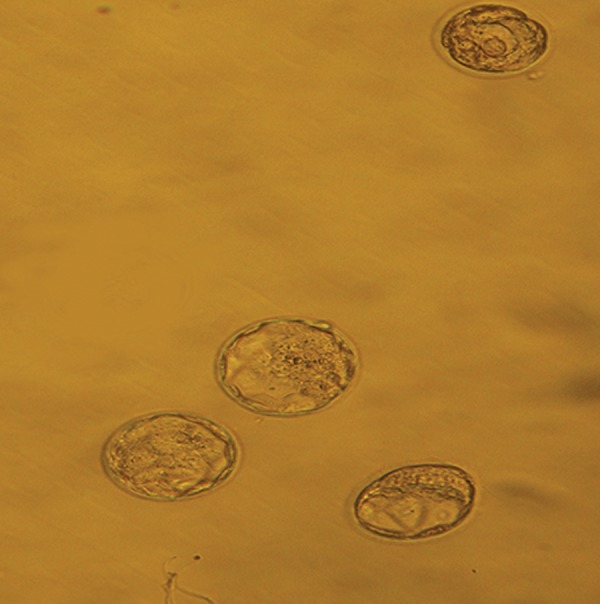
Blastocyst embryos in control group (×200).

## Discussion

AAS alter the function of the hypothalamicpituitary- gonadal (HPG) axis that results in affecting the target reproductive tissues. Acting on the hypothalamus, circulating testosterone regulates the gonadotropin-releasing hormone (GnRH) release. Introducing exogenous AAS to the body increases androgen levels that cause a decrease in luteinizing hormone (LH) release from the pituitary, resulting in the suppression of endogenous testosterone production (22, 23), which is found to be reversible (12). ROS induced damage is major factors in male infertility (24, 25). In the last decade, numerous evidences have been gathered about etiology of oxidative stress (OS) in male infertility (26, 27). Our findings indicated that OXM causes a significant decrease in catalase activity and also total antioxidant concentration. The total antioxidant potential is a measure of the individual’s ability to cope with or prevent a state of OS. It is noted that a state of disequilibrium is the rate of generation of ROS that overwhelms an individual’s ability to remove them (28). Our findings also found that OXM reduces the fertilization rate and percentage of blastocysts, but increases percentage of arrested embryos which is probably due to the effects of OS system. MacLeod (29), observed that human spermatozoa produces ROS, i.e. highly reactive derivatives of oxygen such as H_2_O_2_, and have harmful effects on different sperm functions. ROS involvement in etiology of male infertility is now well-established (30, 31). Since sperm functions are extremely sensitive to ROS -because of high content of polyunsaturated fatty acids (PUFA) and limited ability of DNA repairthe role of ROS and OS in human sperm function and pathophysiology of male infertility have been intensively investigated (32, 33). Very recently, scientists revealed that ROS not only affects the fertilization process negatively, but also raises serious questions about the health and wellbeing of the progeny since the male germ cell is exposed to high oxidants concentrations (34, 35). Furthermore the concept of OS causing damage to spermatozoa by impairing various functions of sperm such as motility, acrosome reaction or DNA integrity is widely accepted nowadays (26, 36, 37). Several studies reported positive effects of RJ which is a homogeneous substance secreted by worker honey bees for feeding young larvae and the adult queen bee on animal reproduction (38). The physical and chemical properties of RJ have been described in detail elsewhere (39, 40). Intramuscular or oral administration of RJ has proven to be effective in improving estrus responses and pregnancy rate (41). The results of this study indicate that RJ increases catalase activity and total antioxidant capacity. Antioxidants including the enzymes superoxide dismutase, catalase and glutathione peroxidase that are constantly produced during normal metabolic and physiological processes (42) counteract with the deleterious effects of ROS mammalian spermatozoa with rich polyunsaturated fatty acids are very susceptible to ROS attack which results in decreased sperm motility, axonemal damage, decreased sperm viability, and increased midpiece morphology defects with deleterious effects on sperm capacitation and acrosome reaction that is presumably due to rapid loss of intracellular ATP (43). The key mechanism of this ROS-induced sperm damage is considered to be lipid peroxidation of sperm membrane that leads to infertility (44). Several studies using various antioxidants alone or in combination with others have shown a significant reduction in seminal ROS levels (45, 46) and improvement in sperm count and motility (47, 48), while other studies have found contrary results (49, 50). We also found that RJ increases fertility-related parameters such as fertilization rate and blastocysts percentage and also reduces percentage of arrested embryos which is probably due to its effect on the total antioxidant capacity. Antioxidants levels in seminal plasma of infertile men are significantly low (51), Renard et al. (52) reported that intravaginal administration of Egyptian bee honey and RJ might be a reasonable and effective method to treat infertility caused by asthenozoospermia. Amino acid content of both honey and RJ may play a main role in fertility either by enhancing acrosome reaction and sperm motility or by improving fertilization. Also sperm motility may be further enhanced by the short chain fatty acids specific to RJ, especially 10-hydroxy2-decenoic acid (45).

## Conclusion

It can be concluded that with the decline in total antioxidant capacity, OXM reduces fertility. Due to beneficial biological properties of its components, RJ showed to increase antioxidant capacity; therefore, it prevents embryo toxicity manifested by OXM when given after OXM administration. As the result, RJ with its antioxidant properties increases fertility.
